# A new deep branch of eurasian mtDNA macrohaplogroup M reveals additional complexity regarding the settlement of Madagascar

**DOI:** 10.1186/1471-2164-10-605

**Published:** 2009-12-14

**Authors:** François-X Ricaut, Harilanto Razafindrazaka, Murray P Cox, Jean-M Dugoujon, Evelyne Guitard, Clement Sambo, Maru Mormina, Marta Mirazon-Lahr, Bertrand Ludes, Eric Crubézy

**Affiliations:** 1 CNRS FRE 2960, Laboratoire d'Anthropobiologie, Université de Toulouse, Toulouse III Paul sabatier, 37 allées Jules Guesde, 31073 Toulouse cedex 3, France; 2 Center for Archaeological Sciences, Katholieke Universiteit Leuven, Celestijnenlaan 200E, 3001 Heverlee, Belgium; 3 Institute of Molecular BioSciences, Allan Wilson Centre for Molecular Ecology and Evolution, and the Bio-Protection Centre, Massey University, Palmerston North 4442, New Zealand; 4 Ecole Normale Supérieure, Université de Toliara, Toliara, Madagascar; 5 Leverhulme Centre for Human Evolutionary Studies, Henry Wellcome Building, Fitzwilliam Street, University of Cambridge, Cambridge CB2 1QH, UK; 6 Laboratoire d'Anthropologie Moléculaire, Institut de Médecine Légale, 11 rue Humann, 67085 Strasbourg, France

## Abstract

**Background:**

Current models propose that mitochondrial DNA macrohaplogroups M and N evolved from haplogroup L3 soon after modern humans left Africa. Increasingly, however, analysis of isolated populations is filling in the details of, and in some cases challenging, aspects of this general model.

**Results:**

Here, we present the first comprehensive study of three such isolated populations from Madagascar: the Mikea hunter-gatherers, the neighbouring Vezo fishermen, and the Merina central highlanders (*n *= 266). Complete mitochondrial DNA genome sequences reveal several unresolved lineages, and a new, deep branch of the out-of-Africa founder clade M has been identified. This new haplogroup, M23, has a limited global distribution, and is restricted to Madagascar and a limited range of African and Southwest Asian groups.

**Conclusions:**

The geographic distribution, phylogenetic placement and molecular age of M23 suggest that the colonization of Madagascar was more complex than previously thought.

## Background

The dominant and widely accepted model of modern human origins proposes that our species originated in Africa ~150,000 years ago (kyr), and after environmental and/or cultural changes, emerged into Eurasia ~85-55 kyr along the Indian Ocean coast toward Australasia (i.e., the Southern Dispersal Route). In terms of human mitochondrial DNA (mtDNA) patterns, this dispersal apparently occurred relatively soon after the appearance of macrohaplogroup L3 in Africa (~85 kyr). The two non-African lineages (macrohaplogroups M and N) diverged shortly afterwards, either just after modern humans left Africa [1-6], or perhaps within Africa slightly earlier, as suggested by an ongoing debate surrounding the early geographical origin of macrohaplogroup M [7-12]. However, beyond this broad-scale view, the settlement patterns of many individual regions are still poorly understood, although some of them are key areas for investigating our species' recent history - either because of their location (e.g., remote and/or close to major dispersal routes), or because they contain isolated or relict populations (e.g., Australia, India, Indonesia). This is the case for Madagascar. The favoured settlement model suggests that the first human groups to reach the island did so extremely recently, around 1.5-2 kyr, when there is clear archaeological evidence of human occupation [13,14]. Furthermore, the genetic, cultural, and linguistic characteristics of the Malagasy indicate that people from both Africa, and Island Southeast Asia played a major role in the colonization of the island, ultimately resulting in a population genetically and linguistically admixed from African and Southeast Asian sources [15-20]. Still, major issues remain unresolved regarding the origin and relative contributions of each founder population to the extant Malagasy gene pool.

The earliest archaeological evidence on the island is controversial. Hippopotamus bones with cut-marks and evidence of human processing from iron tools have been found in the Mikea Forest, in Madagascar's Southwest, dating to ~2 kyr [21]. Later archaeological sites, now containing pottery, have been variously dated from the 4^th ^to the 8^th ^centuries AD. Therefore, the island seems to have been visited at least intermittently by Africans prior to the arrival of Austronesian-speaking maritime travellers from Island Southeast Asia sometime around the 7^th ^or 8^th ^centuries AD [18,19,22-25]. This settlement pattern is further supported by dated faunal extinctions, as well as palaeoenvironmental evidence of deforestation indicated by a decrease in tree pollen and an increase in small charcoal pieces in soil sediments [14,24,26].

The ethnographic evidence is equally complex. All Malagasy today speak a Malayo-Polynesian language, also called Malagasy, which is most closely related to a language spoken in the Barito River basin of Southeast Borneo, Indonesia [18,19,22]. Malagasy contains a number of loan words of African Bantu origin, but these have apparently been borrowed from the Swahili/Sabaki group of languages, and thus form part of the cultural exchange that took place during more recent Indian Ocean trade [22,27]. However, oral tribal traditions suggest the earlier presence of a people called Vazimba, who spoke a non-Malagasy language. Pockets of people still known as Vazimba exist among the island's fishermen, and their non-Malagasy lexicon has also been argued to be of Bantu origin [23,26]. Furthermore, two groups of hunter-gatherers still live on the island - the Beosy and the Mikea, who inhabit the forests of Southwestern Madagascar, and who were recognised as having African affinity as early as the 16^th ^century [24,26].

This paper presents the first comprehensive study of the mtDNA diversity of three Malagasy speaking groups, the Mikea hunter-gatherers, the neighbouring Vezo fishermen, and the Merina central highlanders, and reveals new details regarding the early period of Madagascar's complex history.

## Results and Discussion

Analysis of mtDNA from 266 Malagasy individuals (Table 1) is broadly consistent with previous genetic studies [15-17,20]. We see a combination of Southeast Asian and African lineages that are likely to trace back to the initial settlement of the island around the 7^th ^century AD. However, our results based on complete mitochondrial genomes also revealed the presence of five novel mtDNA lineages that cluster into a previously uncharacterized clade whose geographic distribution seems to be restricted to the island of Madagascar (Additional files 1 and 2). The age estimates for this clade and its main sub-branches are shown in Table 2.

**Table 1:** Inferred frequencies of mtDNA haplogroups in three Malagasy populations: Merina, Vezo, and Mikea.

Malagasy population	mtDNA haplogroups							
	
	L4, L3, L2, L1	M23	M7c3	E1a1a	M46	M*	F3b	B4a1a1a
Mikea (127)	71	**1**	8	13	3	9	5	17
Vezo (101)	47	**4**	9	5	5	3	6	22
Merina (38)	13	**0**	0	4	2	0	0	19
Total (266)	131	**5**	17	22	10	12	11	58

Note. Sequence classification into mtDNA haplogroups was based on accepted nomenclature as detailed in the Materials and Methods section. M* include lineages that were not complete sequenced and thus could not be assignated to currently published haplogroups.

**Table 2:** Molecular dates estimated for the TMRCA and founder of Malagasy haplogroups M23, M23a and M23b from coding region information.

	Coding Region nt577-nt16022								Synonymous Mutations						
	
Haplogroup	n	ρ	σ	Mutation rate	TMRCA	95% CI	Founder age	95% CI	ρ	σ	Mutation rate	TMRCA	95% CI	Founder age	95% CI
M23	5	1.8	0.6	(1)	9,300	3,200-15,300	65,800	49,700-81,900	1.2	0.49	(2)	8,100	1,600-14,600	62,200	44,200-80,200
									1.2	0.49	(3)	9,400	1,900-17,000	72,500	51,500-93,500
M23a	1	2	1.41	(1)	10,300	0-24,400			1	1	(2)	6,800	0-20,000		
									1	1	(3)	7,600	0-23,300		
M23b	4	0.75	0.43	(1)	3,900	0-8,200			0.25	0.25	(2)	1,700	0-5,000		
									0.25	0.25	(3)	2,000	0-5,800		

Mutation rate references: (1) One substitution per 5,139 years [56]; (2) One synonymous transition per 6,764 years [36]; (3) One synonymous mutation (transition or transversion) per 7,884 years [35]. Founder Age: ρ = 12,8; σ = 1.6 with the Mishmar and colleagues approach [56], and ρ = 9.2; σ = 1.36 with the Kivisild and colleagues [36] and Soares and colleagues [35] approaches. TMRCA, 95% confidence intervals (CI), and founder age are given in years.

**Figure 1:**
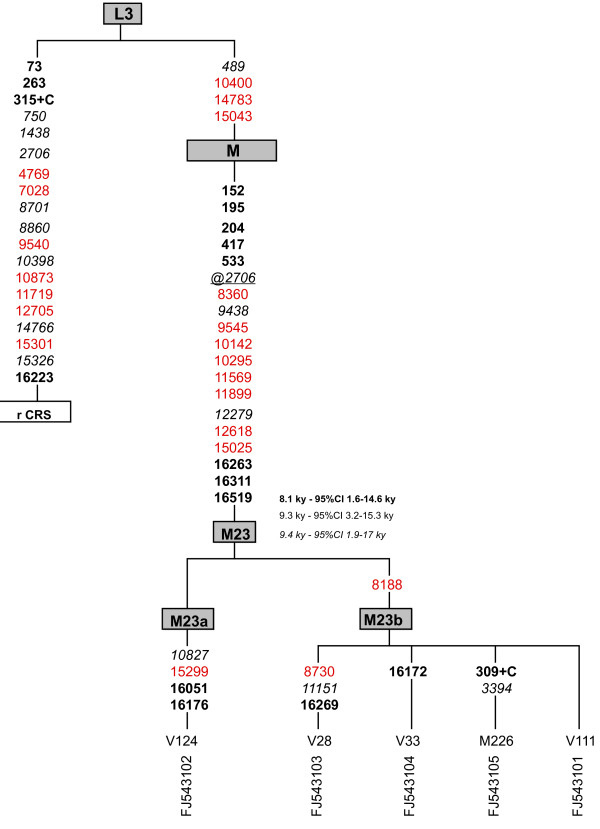
**Phylogenetic tree constructed from complete mtDNA sequences for five Malagasy individuals**. M and V represents the Mikea and Vezo populations, respectively. Mutations were scored relative to the rCRS [42]. Numbers along links refer to nucleotide positions. A plus sign (+) indicates an insertion. Recurrent mutations in the phylogeny are underlined. The prefix "@" indicates back mutations. Control region mutations are in bold, synonymous transitions are shown in red, and non-synonymous mutations are listed in italic. TMRCA estimates were calculated as in [35,36,56], presented in italic, bold and regular font, respectively, and are presented in units of thousands of years before present.

Of the five novel lineages one was found among the Mikea hunter-gatherers (at a frequency <1%) and four among the Vezo fishermen (at a frequency of ~4%) (table 1, highlighted). Comparative phylogenetic analysis of worldwide mtDNA genomes confirmed the clustering of these five lineages into a deep-rooted branch within macrohaplogroup M, which we name M23. This new branch carries all the diagnostic polymorphisms of macrohaplogroup M as well as a substantial series of mutations that separates it from the root of macrohaplogroup M (Figure 1). Haplogroup M23 is characterized by 11 coding region mutations (*viz*. 2706-8360-9438-9545-10142-10295-11569-11899-12279-12618-15025) and 8 control region mutations (*viz*. 152-195-204-417-533-16263-16311-16519) (Figure 1). The absence of a polymorphism at 2706 in the five M23 carriers indicates a back mutation, and we consider this position another basal polymorphism of haplogroup M23. Haplogroup M23 splits into two branches, M23a and M23b. The former is represented by one individual from the Vezo group whilst the latter, defined by a substitution at np 8188 encompasses all the remaining Vezo lineages and is also present in the Mikea.

So far we found no convincing association of M23 with any known M branches. None of the diagnostic coding region mutations of M23 overlaps with the diagnostic markers in other M haplogroups that emerge directly from the root of macrohaplogroup M (see van Oven and Kayser [28]). While some control region mutations (152, 195, 16311, 16263 and 16519) are shared by other deep-rooted M haplogroups (e.g., M1, M28, M29 and M46), these positions are known to be recurrent and cannot be safely considered as linking M23 to other haplogroups within macrohaplogroup M. This confirms the robustness of our phylogenetic reconstruction, and the basal position of M23 within M.

More detailed examination of the phylogeny, geographic distribution, and molecular dating of the M23 lineage reveals three further key points:

(1) As noted before, the position of M23 at the root of macrohaplogroup M indicates that M23 is a deep branch of the human mtDNA phylogeny. The length of the M23 branch suggests either strong genetic drift effects or that this cluster may encompass further branches yet to be identified. Indeed, a relatively small proportion of mtDNA variation has been surveyed in the putative areas of origin of M23. Therefore more extensive sampling is needed to refine the overall geographic distribution and branching structure of this clade, However, the fact that this clade has no specific link to other known branches within macrohaplogroup M suggests a deep-rooted ancestry, possibly tracing back to the Out of Africa event. Such a deep root is also shared with many other lineages that emerged independently from the root of macrohaplogroup M. These lineages are especially prevalent in South Asia [2,29-31]. This general pattern has been interpreted as supporting the view of a rapid dispersal of modern humans at the time of the out-of-Africa exodus, followed by a long period of isolation resulting in non-overlapping distributions of derived M haplogroups in relict or isolated populations/regions along the dispersal route. Thus, our results suggest that the Mikea hunter-gatherers and Vezo fishermen of Madagascar descend, if only in very small part (≤4%), from one such deep-rooted, isolated population.

(2) M23 lineages have an extremely restricted geographic distribution. A survey of all complete mtDNA sequences reported in the literature (>6,700 sequences; http://www.phylotree.org/) could not detect M23 sequences anywhere outside Madagascar. Moreover, the screening of control region polymorphisms that are diagnostic for M23 against a larger global panel of mtDNA control region variants confirmed that the M23 control region motif is indeed rare, as only four individuals shared the 13 control region diagnostic mutations for M23 (Additional file 1). Although comparative analysis based only on the first hypervariable sequence (HVS1) reveals a few more individuals that share the four HVS1 mutations of M23 (16223, 16263, 16311 and 16519), these nucleotide positions are known to be fast-mutating and recurrent, and consequently cannot be considered diagnostic of haplogroup M23 (Additional files 1, 2 and 3). Interestingly, three of the four individuals sharing the 13 control region mutations for M23 are African Americans who are likely to trace their ancestry to sub-Saharan Africans, although no M23 carriers have been detected on mainland Africa itself (Additional files 2 and 3). The fourth individual is from the Arabian Peninsula (Dubai, United Arab Emirates), a region placed in Southwest Asia which has a long history of interactions with Africa, probably dating back to the dispersal of modern human along the southern dispersal route [3,4,6]. The modern population of Dubai has a genetic composition strongly influenced by female-mediated gene flow from sub-Saharan Africa, as well as migration from South Asian populations [32], which have the highest observed levels of basal M lineages [2,29,31,33]. Although we have only detected four individuals potentially affiliated to M23, they are likely to descend from an African and/or Southwest Asian source, again placing the origin of M23 somewhere between these two regions. Unfortunately, lacking genealogical records for these four individuals, we cannot confirm their maternal African origin, and without additional mtDNA coding region information, the link with African populations remains highly speculative. However, if confirmed, this finding would suggest that the origin and dispersal of M23 lineages is restricted to the circum-Arabia/northwestern Indian Ocean regions.

(3) Despite the limitations of molecular dating [34], the estimated founder age of macrohaplogroup M using the M23 branch considered alone is 62-73 kyr (95% confidence interval, 44-94 kyr) (Table 2). This conforms to the revised age estimate of macrohaplogroup M [35], and is slightly older than the proposed date for the dispersal of anatomically modern humans from Africa, as well as the population expansion accompanying it [2-4,33,36]. The time to the most recent common ancestor (TMRCA) of M23 has been estimated at 9.4 kyr (95% confidence interval: 1.9-17 kyr) using a recently improved control region mutation rate [35] (Table 2), in broad agreement with dates obtained using previous coding region mutation rates (Table 2). Considering that a demographic expansion may predate a geographic one, it is worth noting that the lower age estimates of M23, and especially of its subclade M23b, fall clearly within the Holocene (1.7-3.9 kyr; 95% confidence interval, 0-8.2 kyr). Although this is broadly consistent with a late Holocene date for the initial settlement of Madagascar [14,18,19,21-25] and the concomitant demographic/geographic expansions, the large confidence intervals add uncertainty to the dispersal date of M23 and leave open the possibility that this rare lineage may represent an early pre-Austronesian expansion into Madagascar.

The presence of the M23 clade among the Malagasy Vezo fishermen and Mikea hunter-gatherers provides additional mtDNA evidence upon which a better picture of the colonization of Madagascar can be built. However, open questions remain, including the geographic origin of M23, and the time and mode of its spread into Madagascar. These outstanding issues can only be partially investigated with the currently available data. The M23 lineage is not present in any of the putative parental populations of the Malagasy (Africans and Island Southeast Asians), suggesting either its absence from these populations, or that it is so exceedingly rare there that it has not yet been detected [17,37-40] (Additional files 1 and 2). Indeed, relative to their genetic diversity, Africans and Southeast Asians have not been widely sampled, although Borneo (the likely source of the Austroensian expansion into Madagascar) has been relatively well surveyed, and a high number of published mtDNA sequences (n = 157) is currently available from this area [38]. Nonetheless, M23 lineages have not been identified in this region. Even if M23 is as rare in Borneo as it is in Madagascar (1.9%), the probability of it being detected there is high: *P*(M23 | n, freq) = 0.95. However, the extreme population structure of Indonesia [41] may mean that M23 is restricted to populations that have not yet been sufficiently sampled, or at all.

An alternative hypothesis is that the M23 motif developed *in situ *in Madagascar, either completely or partially. If this is the case, a pre-M23 lineage should have evolved more or less in isolation within the founder population that later participated in the colonization of Madagascar.

The identification of four individuals of African and Southwest Asian origin who share the 13 diagnostic control region mutations for M23 pinpoints these regions as potential sources for M23. Whilst, the data does not allow us to make clear phylogeographic inferences regarding M23 origin, our results may provide some evidence of ancient contacts across the Indian Ocean involving Africa, Madagascar and South Asia. The deep-rooted topology of M23 and its age estimate coupled with its very restricted distribution within Madagascar, makes unlikely its presence in the island as a result of recent contacts, and is more in agreement with the patterns of human contacts across the Arabian Sea and the Indian Ocean, which pre-dated the Austronesian expansion into Madagascar [24,27].

Whilst more extensive screening of the putative parental populations in Africa and South Asia will help to ascertain the geographic origin and distribution of M23, our initial examination of Malagasy mtDNA diversity suggests that the origin of M23 lineages may be found in the circum-Arabia/northwestern Indian Ocean regions and that their arrival to Madagascar may pre-date the Austronesian settlement of the island. This lends support to oral tribal traditions stressing the earlier presence of non-Malagasy speakers (e.g. Vazimba; [23,24,26]) and re-emphasizes the importance and complexity of the circum-Arabia and Indian Ocean corridor since the late Pleistocene.

## Conclusion

The finding of a new deep branch of the out-of-Africa founder M, named as M23, in fishermen and hunter-gatherers from Southwestern Madagascar raises many questions regarding both the clade's origin and its role in the settlement of Madagascar. Extant data cannot provide unequivocal evidence for the origin of M23, although the current distribution of macrohaplogroup M points to Southeast Asia as the most likely source region. Additional archaeological surveys, population sampling from South Asia and East Africa/Madagascar, and further phylogeographic analyses are necessary to ascertain the exact time and place of origin of this clade, as well as its geographic dispersal. However, this novel mtDNA branch already provides a new suite of diagnostic markers to expand the search for its geographical and temporal origin.

## Methods

### Population samples

The samples analyzed in this study were taken from our Malagasy assemblage, which was collected in field seasons 2007-2008. The samples were obtained with informed consent, and were approved by Human Subjects' Ethics Committees in Madagascar, and at the University of Toulouse, France. Buccal cells and peripheral blood were sampled from unrelated individuals, and stored in EDTA Vaccutainer tubes. Subjects were surveyed for language affiliation, current residence, familial birthplaces, and a short genealogy of four generations to establish regional ancestry. A total of 266 DNA samples were analyzed from three ethnic groups: 127 Mikea (hunter-gatherers located in the Southwest), 101 Vezo (semi-nomadic fishermen also located in the Southwest), and 38 Merina (highlanders from central Madagascar).

### DNA extraction, amplification and sequencing

DNA was extracted using a standard phenol-chloroform method, ethanol precipitated, and stored in Tris EDTA at -20°C until further use. Analysis proceeded in three phases.

First, we amplified and sequenced hypervariable segments 1 and 2 (HVS1 and HVS2) of the mitochondrial DNA control region using primers: L15973 (5'-AACTCCACCATTAGCACCCA-3'), and H296 (5'-TCTGTAGTATTGTTTTTTAAAGG-3'). Samples were sequenced by the Genopole Toulouse Sequencing Service http://www.genotoul.fr/ on an ABI PRISM 3730 Genetic Analyzer. Sequences were edited and aligned against the revised Cambridge reference sequence (rCRS) [42] using BioEdit 7.0.9 [43]. Deviations from the rCRS were confirmed by checking electropherograms manually.

Second, for samples not definitively assigned to a known haplogroup from HVS1 and HVS2 sequence variation alone, we screened for mutations defining macrohaplogroups L3 (-10871 *Mnl*II), M (+10397 *Alu*I; +10394 *Dde*I), and N (-10397 *Alu*I; -10394 *Dde*I). Then, depending on the presence or absence of these sites, additional RFLPs were surveyed to identify other haplogroups common in Southeast Asia: E (-7598 *Hha*I); E1 (+10834 *Mse*I); D (- 5176 *Alu*I); M7 (+9820 *Hinf*I); M7c3 (+3606 *Sau96 *I); M7c1 (+ 3882 *Bsa*I), all within macrohaplogroup M; and F (+10306 *Bspm*I); F3 (+10319 *Tsp509 *I) and B4a1a (+6719 *Nla*III), all within macrohaplogroup N. To confirm affiliation of samples to haplogroup B, the 9-base pair intergenic region V deletion [44] was amplified using primers L8196 and H8297 [45].,

Third, 5 representative samples from the 17 samples remaining with uncertain phylogenetic status (i.e., that could not be assigned to currently recognized haplogroups) were selected for complete mtDNA sequencing by the Genomic Analysis Technology Core (GATC) at the University of Arizona http://uagc.arl.arizona.edu/ . The 12 remaining samples were not completely sequenced, and consequently we do not know whether they belong to M23. Using twenty-eight pairs of primers, overlapping fragments of forward and reverse DNA strands were amplified and sequenced. Sequences were edited and aligned as described above. The five complete mtDNA sequences have been deposited in the GenBank database (Accession Numbers: FJ543101-FJ543105).

We utilized strict quality control to avoid errors and artefacts (e.g., base shift, reference bias, phantom mutations, errors in base scoring, and artifactual recombination) as proposed by Bandelt et al. [46]: (i) each base pair was determined with both forward and reverse primers ensuring overlapping sequencing of both strands; (ii) we rechecked all sequence variations by manual observation of sequence electropherograms; (iii) as well as checking for any incongruence with results obtained from PCR. Moreover, we checked that all sequence variations observed have previously been reported http://www.mitomap.org/, and that haplogroup and sub-haplogroup motifs were fully represented.

### Statistical analysis

#### Geographic distribution

To estimate the geographic distribution of M23 lineages, we compared the five Malagasy M23 complete mtDNA sequences with more than 6,700 complete mtDNA sequences compiled by van Oven and Kayser http://www.phylotree.org/; [28]), a dataset that contains all of the complete mtDNA genomes available to date. However, as whole genome sequences are rare for some regions and populations, especially those known to have high genetic diversity (e.g., Eastern Africa, Southern India, Indonesia), we also performed a comparative analysis using partial or entire mtDNA control region sequences. (Note that we discarded indels at position 309, 315, 573, and 16193). This comparison was made by screening an in-house database of 43,849 HVS1 sequences collated from the literature, as well as several web-based mtDNA databases: (1) DDBJ/EMBL/GENBANK international nucleotide sequence database; (2) mtDNAmanager http://mtmanager.yonsei.ac.kr/[47]; (3) The EMPOP database http://www.empop.org/; [48]); (4) the Genographic Project Open Resource Mitochondrial DNA database [49]; and (5) HvrBase++ http://www.hvrbase.org; [50]. This comparative analysis allowed us to survey most of the HVS sequences published thus far. However, although providing larger numbers of samples for analysis, this comparison is limited by higher rates of homoplasy and back mutation in the mtDNA hypervariable sequences compared to coding regions. This can lead to evolutionary convergence, and therefore confound unrelated sequences that are "identical by state" (IBS) as opposed to "identical by descendent" (IBD) [49].

#### Phylogeny reconstruction

Sequence classification into mtDNA haplogroups was based on accepted nomenclature (e.g., [1,9,29-31,36,37,40,51-53]; http://www.phylotree.org/; http://mtmanager.yonsei.ac.kr/). The phylogenetic tree was reconstructed from median-joining networks rooted to L3 using Network 4.2.0.1 http://www.fluxus-engineering.com/[54]. The tree was checked manually to resolve homoplasies.

#### Molecular dating

TMRCA estimates were calculated using the rho (ρ) statistic and its standard deviation (SD) [55] with three previously described mutation rates based on coding region mutations. Two mutation rates were calculated using estimated substitution rates; one of 3.5 × 10^-8 ^mutations/site/year for protein-coding synonymous changes, which yields 6,764 years per synonymous transition [36], and another of one synonymous mutation (including transitions and transversions) every 7,884 years from the recently improved mitochondrial molecular clock published by Soares and colleagues [35]. Data from the MAMMAG website http://mammag.web.uci.edu/bin/view/Main/WebHome was used to identify synonymous transitions. The third mutation rate was based on substitution changes for the entire coding region (1.26 × 10^-8 ^mutation/site/year), which is equivalent to 5,139 years per mutation [56] between positions 577 and 16023 of the rCRS [42]. All three rates were calibrated by comparison to chimpanzee sequences using a divergence time between human and chimpanzee mtDNAs of 6.5 million years. Dates estimated from synonymous changes are likely to be more robust, as these changes are mostly selectively neutral [36]. It is worth noting that due to the ongoing debate regarding the true mutation rate [36,57], and the limitations of the rho (ρ) statistic method of molecular dating [34], the conversion of age estimates in mutations into ages in years and the estimation of associated error values are to be considered approximations only. These dates should be interpreted cautiously.

## Authors' contributions

JMD, EC, BL, FXR, and CS designed research; HR and EG performed research; FXR, HR, MPC, and EC analyzed data; FXR, HR, MPC, MM and MML wrote the paper. All authors read and approved the final manuscript.

## Supplementary Material

Additional file 1 **Distribution of diagnostic mtDNA control region polymorphisms in haplogroup M23 (73G 152C 195C 204C 263G 315.1C 417A 489C 533G 16223T 16263C 16311C 16519C) from available databases**. The table displays the occurrence of the mtDNA control region diagnostic polymorphisms of haplogroup M23 in six mtDNA databases (≤1 mismatch). Click here for file

Additional file 2 **Distribution of HVS1 polymorphisms defining haplogroup M23 within sub-Saharan African populations**. The table displays the occurrence of the M23 HVS1 motif in 7,262 sub-Saharan Africans. Click here for file

Additional file 3 **Reference list for the supplemental citations in Additional files**1**and**2. Click here for file
