# Supplementary material for: A new deep branch of eurasian mtDNA macrohaplogroup M reveals additional complexity regarding the settlement of Madagascar
Source: BMC Genomics. 2009 Dec 14;10:605. doi: 10.1186/1471-2164-10-605 (PMC2808327; doi:10.1186/1471-2164-10-605)
Supplement: Additional file 3 — Reference list for the supplemental citations in Additional files1and2. [file 1471-2164-10-605-S3.DOC]

1. Batini C, Coia V, Battaggia C, Rocha J, Pilkington MM, Spedini G, Comas D, Destro-Bisol G, Calafell F: **Phylogeography of the human mitochondrial L1c haplogroup: genetic signatures of the prehistory of Central Africa**. *Mol Phylogenet Evol* 2007, **43**(2):635-644.

2. Behar DM, Villems R, Soodyall H, Blue-Smith J, Pereira L, Metspalu E, Scozzari R, Makkan H, Tzur S, Comas D *et al*: **The dawn of human matrilineal diversity**. *Am J Hum Genet* 2008, **82**(5):1130-1140.

3. Beleza S, Gusmao L, Amorim A, Carracedo A, Salas A: **The genetic legacy of western Bantu migrations**. *Hum Genet* 2005, **117**(4):366-375.

4. Berniell-Lee G, Plaza S, Bosch E, Calafell F, Jourdan E, Cesari M, Lefranc G, Comas D: **Admixture and sexual bias in the population settlement of La Reunion Island (Indian Ocean)**. *Am J Phys Anthropol* 2008, **136**(1):100-107.

5. Brandstatter A, Peterson CT, Irwin JA, Mpoke S, Koech DK, Parson W, Parsons TJ: **Mitochondrial DNA control region sequences from Nairobi (Kenya): inferring phylogenetic parameters for the establishment of a forensic database**. *Int J Legal Med* 2004, **118**(5):294-306.

6. Brehm A, Pereira L, Bandelt HJ, Prata MJ, Amorim A: **Mitochondrial portrait of the Cabo Verde archipelago: the Senegambian outpost of Atlantic slave trade**. *Ann Hum Genet* 2002, **66**(Pt 1):49-60.

7. Castri L, Tofanelli S, Garagnani P, Bini C, Fosella X, Pelotti S, Paoli G, Pettener D, Luiselli D: **mtDNA variability in two Bantu-speaking populations (Shona and Hutu) from Eastern Africa: Implications for peopling and migration patterns in sub-Saharan Africa**. *Am J Phys Anthropol* 2009.

8. Cox MP: **Genetic Patterning at Austronesian Contact Zones**. Ph.D. thesis, University of Otago, New Zealand, 2003

9. Cerny V, Salas A, Hajek M, Zaloudkova M, Brdicka R: **A bidirectional corridor in the Sahel-Sudan belt and the distinctive features of the Chad Basin populations: a history revealed by the mitochondrial DNA genome**. *Ann Hum Genet* 2007, **71**(Pt 4):433-452.

10. Cerny V, Hajek M, Cmejla R, Bruzek J, Brdicka R: **mtDNA sequences of Chadic-speaking populations from northern Cameroon suggest their affinities with eastern Africa**. *Ann Hum Biol* 2004, **31**(5):554-569.

11. Chen YS, Olckers A, Schurr TG, Kogelnik AM, Huoponen K, Wallace DC: **mtDNA variation in the South African Kung and Khwe-and their genetic relationships to other African populations**. *Am J Hum Genet* 2000, **66**(4):1362-1383.

12. Coelho M, Sequeira F, Luiselli D, Beleza S, Rocha J: **On the edge of Bantu expansions: mtDNA, Y chromosome and lactase persistence genetic variation in southwestern Angola**. *BMC Evol Biol* 2009, **9**:80.

13. Coia V, Destro-Bisol G, Verginelli F, Battaggia C, Boschi I, Cruciani F, Spedini G, Comas D, Calafell F: **Brief communication: mtDNA variation in North Cameroon: lack of Asian lineages and implications for back migration from Asia to sub-Saharan Africa**. *Am J Phys Anthropol* 2005, **128**(3):678-681.

14. Destro-Bisol G, Coia V, Boschi I, Verginelli F, Caglia A, Pascali V, Spedini G, Calafell F: **The analysis of variation of mtDNA hypervariable region 1 suggests that Eastern and Western Pygmies diverged before the Bantu expansion**. *Am Nat* 2004, **163**(2):212-226.

15. Destro-Bisol G, Donati F, Coia V, Boschi I, Verginelli F, Caglia A, Tofanelli S, Spedini G, Capelli C: **Variation of female and male lineages in sub-Saharan populations: the importance of sociocultural factors**. *Mol Biol Evol* 2004, **21**(9):1673-1682.

16. Dubut V, Murail P, Pech N, Thionville MD, Cartault F: **Inter- and extra-Indian admixture and genetic diversity in reunion island revealed by analysis of mitochondrial DNA**. *Ann Hum Genet* 2009, **73**(Pt 3):314-334.

17. Ely B, Wilson JL, Jackson F, Jackson BA: **African-American mitochondrial DNAs often match mtDNAs found in multiple African ethnic groups**. *BMC Biol* 2006, **4**:34.

18. Gonder MK, Mortensen HM, Reed FA, de Sousa A, Tishkoff SA: **Whole-mtDNA genome sequence analysis of ancient African lineages**. *Mol Biol Evol* 2007, **24**(3):757-768.

19. Gonzalez AM, Cabrera VM, Larruga JM, Tounkara A, Noumsi G, Thomas BN, Moulds JM: **Mitochondrial DNA variation in Mauritania and Mali and their genetic relationship to other Western Africa populations**. *Ann Hum Genet* 2006, **70**(Pt 5):631-657.

20. Graven L, Passarino G, Semino O, Boursot P, Santachiara-Benerecetti S, Langaney A, Excoffier L: **Evolutionary correlation between control region sequence and restriction polymorphisms in the mitochondrial genome of a large Senegalese Mandenka sample**. *Mol Biol Evol* 1995, **12**(2):334-345.

21. Rando JC, Pinto F, Gonzalez AM, Hernandez M, Larruga JM, Cabrera VM, Bandelt HJ: **Mitochondrial DNA analysis of northwest African populations reveals genetic exchanges with European, near-eastern, and sub-Saharan populations**. *Ann Hum Genet* 1998, **62**(Pt 6):531-550.

22. Horai S, Hayasaka K: **Intraspecific nucleotide sequence differences in the major noncoding region of human mitochondrial DNA**. *Am J Hum Genet* 1990, **46**(4):828-842.

23. Howell N, Elson JL, Turnbull DM, Herrnstadt C: **African Haplogroup L mtDNA sequences show violations of clock-like evolution**. *Mol Biol Evol* 2004, **21**(10):1843-1854.

24. Hurles ME, Sykes BC, Jobling MA, Forster P: **The dual origin of the Malagasy in Island Southeast Asia and East Africa: evidence from maternal and paternal lineages**. *Am J Hum Genet* 2005, **76**(5):894-901.

25. Ingman M, Kaessmann H, Paabo S, Gyllensten U: **Mitochondrial genome variation and the origin of modern humans**. *Nature* 2000, **408**(6813):708-713.

26. Jackson BA, Wilson JL, Kirbah S, Sidney SS, Rosenberger J, Bassie L, Alie JA, McLean DC, Garvey WT, Ely B: **Mitochondrial DNA genetic diversity among four ethnic groups in Sierra Leone**. *Am J Phys Anthropol* 2005, **128**(1):156-163.

27. Kivisild T, Reidla M, Metspalu E, Rosa A, Brehm A, Pennarun E, Parik J, Geberhiwot T, Usanga E, Villems R: **Ethiopian mitochondrial DNA heritage: tracking gene flow across and around the gate of tears**. *Am J Hum Genet* 2004, **75**(5):752-770.

28. Kivisild T, Shen P, Wall DP, Do B, Sung R, Davis K, Passarino G, Underhill PA, Scharfe C, Torroni A *et al*: **The role of selection in the evolution of human mitochondrial genomes**. *Genetics* 2006, **172**(1):373-387.

29. Knight A, Underhill PA, Mortensen HM, Zhivotovsky LA, Lin AA, Henn BM, Louis D, Ruhlen M, Mountain JL: **African Y chromosome and mtDNA divergence provides insight into the history of click languages**. *Curr Biol* 2003, **13**(6):464-473.

30. Krings M, Salem AE, Bauer K, Geisert H, Malek AK, Chaix L, Simon C, Welsby D, Di Rienzo A, Utermann G *et al*: **mtDNA analysis of Nile River Valley populations: A genetic corridor or a barrier to migration?** *Am J Hum Genet* 1999, **64**(4):1166-1176.

31. Maca-Meyer N, Gonzalez AM, Larruga JM, Flores C, Cabrera VM: **Major genomic mitochondrial lineages delineate early human expansions**. *BMC Genet* 2001, **2**:13.

32. Mateu E, Comas D, Calafell F, Perez-Lezaun A, Abade A, Bertranpetit J: **A tale of two islands: population history and mitochondrial DNA sequence variation of Bioko and Sao Tome, Gulf of Guinea**. *Ann Hum Genet* 1997, **61**(Pt 6):507-518.

33. Mishmar D, Ruiz-Pesini E, Golik P, Macaulay V, Clark AG, Hosseini S, Brandon M, Easley K, Chen E, Brown MD *et al*: **Natural selection shaped regional mtDNA variation in humans**. *Proc Natl Acad Sci U S A* 2003, **100**(1):171-176.

34. Monson KL, Miller, K.W.P., Wilson, M.R., Dizinno, J.A., Budowle, B.: **The mtDNA population database: an integrated software and database resource for forensic comparison**. In*.*, vol. 4: Forensic Science Communications, 4, no 2 2002.

35. Pereira L, Macaulay V, Torroni A, Scozzari R, Prata MJ, Amorim A: **Prehistoric and historic traces in the mtDNA of Mozambique: insights into the Bantu expansions and the slave trade**. *Ann Hum Genet* 2001, **65**(Pt 5):439-458.

36. Pinto F, Gonzalez AM, Hernandez M, Larruga JM, Cabrera VM: **Genetic relationship between the Canary Islanders and their African and Spanish ancestors inferred from mitochondrial DNA sequences**. *Ann Hum Genet* 1996, **60**(Pt 4):321-330.

37. Plaza S, Salas A, Calafell F, Corte-Real F, Bertranpetit J, Carracedo A, Comas D: **Insights into the western Bantu dispersal: mtDNA lineage analysis in Angola**. *Hum Genet* 2004, **115**(5):439-447.

38. Quintana-Murci L, Semino O, Bandelt HJ, Passarino G, McElreavey K, Santachiara-Benerecetti AS: **Genetic evidence of an early exit of Homo sapiens sapiens from Africa through eastern Africa**. *Nat Genet* 1999, **23**(4):437-441.

39. Quintana-Murci L, Quach H, Harmant C, Luca F, Massonnet B, Patin E, Sica L, Mouguiama-Daouda P, Comas D, Tzur S *et al*: **Maternal traces of deep common ancestry and asymmetric gene flow between Pygmy hunter-gatherers and Bantu-speaking farmers**. *Proc Natl Acad Sci U S A* 2008, **105**(5):1596-1601.

40. Rosa A, Brehm A, Kivisild T, Metspalu E, Villems R: **MtDNA profile of West Africa Guineans: towards a better understanding of the Senegambia region**. *Ann Hum Genet* 2004, **68**(Pt 4):340-352.

41. Salas A, Richards M, De la Fe T, Lareu MV, Sobrino B, Sanchez-Diz P, Macaulay V, Carracedo A: **The making of the African mtDNA landscape**. *Am J Hum Genet* 2002, **71**(5):1082-1111.

42. Soodyall H, Vigilant L, Hill AV, Stoneking M, Jenkins T: **mtDNA control-region sequence variation suggests multiple independent origins of an "Asian-specific" 9-bp deletion in sub-Saharan Africans**. *Am J Hum Genet* 1996, **58**(3):595-608.

43. Stevanovitch A, Gilles A, Bouzaid E, Kefi R, Paris F, Gayraud RP, Spadoni JL, El-Chenawi F, Beraud-Colomb E: **Mitochondrial DNA sequence diversity in a sedentary population from Egypt**. *Ann Hum Genet* 2004, **68**(Pt 1):23-39.

44. Tishkoff SA, Gonder MK, Henn BM, Mortensen H, Knight A, Gignoux C, Fernandopulle N, Lema G, Nyambo TB, Ramakrishnan U *et al*: **History of click-speaking populations of Africa inferred from mtDNA and Y chromosome genetic variation**. *Mol Biol Evol* 2007, **24**(10):2180-2195.

45. Torroni A, Achilli A, Macaulay V, Richards M, Bandelt HJ: **Harvesting the fruit of the human mtDNA tree**. *Trends Genet* 2006, **22**(6):339-345.

46. Torroni A, Rengo C, Guida V, Cruciani F, Sellitto D, Coppa A, Calderon FL, Simionati B, Valle G, Richards M *et al*: **Do the four clades of the mtDNA haplogroup L2 evolve at different rates?** *Am J Hum Genet* 2001, **69**(6):1348-1356.

47. Trovoada MJ, Pereira L, Gusmao L, Abade A, Amorim A, Prata MJ: **Pattern of mtDNA variation in three populations from Sao Tome e Principe**. *Ann Hum Genet* 2004, **68**(Pt 1):40-54.

48. Vigilant L, Stoneking M, Harpending H, Hawkes K, Wilson AC: **African populations and the evolution of human mitochondrial DNA**. *Science* 1991, **253**(5027):1503-1507.

49. Watson E, Forster P, Richards M, Bandelt HJ: **Mitochondrial footprints of human expansions in Africa**. *Am J Hum Genet* 1997, **61**(3):691-704.

50. Watson E, Bauer K, Aman R, Weiss G, von Haeseler A, Paabo S: **mtDNA sequence diversity in Africa**. *Am J Hum Genet* 1996, **59**(2):437-444.

51. Alshamali F, Brandstatter A, Zimmermann B, Parson W: **Mitochondrial DNA control region variation in Dubai, United Arab Emirates**. *Forensic Sci Int Genet* 2008, **2**(1):e9-10.

52. Tofanelli S, Bertoncini S, Castri L, Luiselli D, Calafell F, Donati G, Paoli G: **On the origins and admixture of Malagasy: new evidence from high-resolution analyses of paternal and maternal lineages**. *Mol Biol Evol* 2009, **26**(9):2109-2124.

53. Behar DM, Rosset S, Blue-Smith J, Balanovsky O, Tzur S, Comas D, Mitchell RJ, Quintana-Murci L, Tyler-Smith C, Wells RS: **The Genographic Project public participation mitochondrial DNA database**. *PLoS Genet* 2007, **3**(6):e104.
